# Home Department

**Published:** 1886-02

**Authors:** 


### HOME DEPARTMENT.

**For Loss of Voice.**—Take white oak bark, boil until the strength is extracted, strain, add vinegar and cayenne pepper. To be gargled frequently.

**To Make Crackers Crisp.**—Crackers that have softened by exposure will become crisp and fresh again by being heated in the oven a few minutes.

**Oyster Omelet.**—Add to a half a cup of cream six eggs beaten very lightly, season with salt and pepper, and pour into a frying-pan with a tablespoonfull of butter, drop in a dozen large oysters, and fry a light brown. Double over, and serve immediately.

**Rye Cakes.**—One pint of rich milk, three eggs and a half teaspooonful of salt. Mix with enough rye meal to make a thin batter. Half fill cups or deep patty pans with the batter and bake for 20 minutes.

**Dressing for Poultry.**—Pour boiling water over stale bread or crackers, and when perfectly soft, add one egg, a small piece of butter, salt and pepper to taste, and nutmeg instead of sage or onions, as the flavor is much finer. Try it.

**Graham Pudding.**—One and one-half cups of Graham flour half a cup of seeded raisins, half a cup of dried currants, half a cup of molasses, half a cup of chopped suet, and half a teaspoonful of soda. Add one egg, salt, and plenty of spice, and steam for two and one-half hours.

**Poached Eggs.**—Nearly fill frying-pan with boiling water; add a little salt and vinegar. Break eggs one at a time into wet sauce; slip from this upon surface of water. Cook slowly three minutes; take up with perforated skimmer; lay carefully upon buttered toast.

**For Chapped Hands, &c.**—Fine almond oil, 1 lb.· simple syrup V. S. P., 4 ozs.; white soft soap, 1 oz.; oil of almond, 1 oz.; oil of bergamut, 1 oz.; oil of cloves, 1 oz. Rub the syrup with the soap until the mixture is homogeneous, then rub in the oil by degrees.

**Worth Knowing.**—Fat of chickens is said by a cake-maker of great experience, to be superior to the finest butter for making the most delicate cake, If the fat of boiled chickens is to be used, cook them without salt, and there will not be the slightest flavor of fowl.

**Soft Boiled Eggs.**—Fresh eggs for invalids who would like them cooked soft, should be put in a pan of boiling water, and set on a part of the stove where they will not boil for several minutes. At the end of that time they will be like jelly, perfectly soft but nicely done, and quite digestible by even weak stomachs.

**The** history of the Roman Empire in the time of Julius Cæsar shows that wheat, as an article of food, combined with fresh outdoor-air life, is capable of producing and sustaining the highest type of physical manhood the world has ever seen. The empire built up and maintained by soldiers whose main article of food was wheat.

**Beef Tea.**—Beef to be used for beef tea should be cut fine or chopped, and then soaked in cold water for two hours, if the time can be spared, and placed upon the fire in the same water. After thorough boiling it should be strained, all the fat carefully removed, and a little salt added. Allow a pint of water to every pound of meat.

**Broiled Potatoes.**—Raw and boiled potatoes are served in this manner: Cut the raw potatoes in thin slices, brush melted butter over them and also over the wire broiler, to prevent their sticking to it; broil them a dark brown; boiled sweet potatoes need to be but slightly broiled—just enough to warm through, and at the same time to show the marks of the broiler.

**Vegetable Soup.**—This will make a fine vegetable soup: Take a three-pint bowl of vegetables of all kinds, cut up very small; boil them in two quarts of water, with a little salt; when done, blend two tablespoonfuls of flour, with a piece of butter the size of an egg, and add a pint of cream or a pint and a half of milk; boil all together and just before serving add the yolks of two eggs mixed with a little cream or milk.

**To Clean Piano Keys.**—When the white pianoforte keys become discolored we should remove the front fall and slip of wood just over them; then lift up each key separately from the front—do not take them out—and rub the keys with a white cloth slightly dampened with cold water, and dry off with a cloth slightly warm. Should the keys be sticky first damp the cloth with a little spirits of wine or gin. Soap washing powder should not be used. It is worth while to keep a supply of ammonia in the household in case we wish to remove finger marks from paint or require to cleanse brushes or greasy pans. A teaspoonful in a basin of warm water will make hair brushes beautifully white; but care must be taken not to let the backs of the brushes dip below the surface. Rinse them with clean warm water and put in a sunny window to dry.

